# Artemisinin: A Panacea Eligible for Unrestrictive Use?

**DOI:** 10.3389/fphar.2017.00737

**Published:** 2017-10-17

**Authors:** Dong-Sheng Yuan, Yan-Ping Chen, Li-Li Tan, Shui-Qing Huang, Chang-Qing Li, Qi Wang, Qing-Ping Zeng

**Affiliations:** ^1^Clinical Pharmacology Institute, Guangzhou University of Chinese Medicine, Guangzhou, China; ^2^Tropical Medicine Institute, Guangzhou University of Chinese Medicine, Guangzhou, China; ^3^Basic Medical Science College, Guangzhou University of Chinese Medicine, Guangzhou, China

**Keywords:** artemisinin, multi-target, wide-spectrum, malarial resistance, restrictive use

## Abstract

Although artemisinin has been used as anti-malarial drug, accumulating evidence on the extended therapeutic potential of artemisinin emerges. Apart from anti-malaria and anti-tumor, artemisinin can also exert beneficial effects on some metabolic disorders, such as obesity, diabetes, and aging-related diseases. However, whether artemisinin should be applied to treatment of the wide-spectrum diseases is debating. Here, we discuss the predisposition of a raised risk of malarial resistance to artemisinin from consideration of the multi-target and non-specific features of artemisinin.

Artemisinin, a sesquiterpene endoperoxide lactone that naturally occurs in the medicinal plant *Artemisia annua* L., has been extracted and manufactured as an anti-malarial drug for decades (Majori, 2004). It may also hold promise for a clinical application in anti-tumor (Efferth, 2017). Mechanistically, artemisinin, with its unique endoperoxide bridge structure, binds to cytosolic and/or mitochondrial targets to interfere with signal transduction and/or affect electron transport in a peroxide-dependent manner. However, it remains unconvinced which and how many cellular components are targeted by artemisinin. Based on their functional changes, the heme-containing enzymes, nitric oxide synthase (NOS) and catalase (CAT), as the targets of artemisinin were clarified from bacteria (Zeng et al., 2011) and tumor cells (Zeng and Zhang, 2011). Actually, artemisinin also interacts with non-heme proteins and many other kinds of proteins. For example, as many as 124 malarial non-heme proteins that covalently bind to artemisinin were successfully identified in the malarial parasite *Plasmodium falciparum* (Wang et al., 2015).

## Artemisinin Targets Cytosolic or Mitochondrial Proteins Involved in Modulating Metabolic Homeostasis

The covalent conjugation of artemisinin with heme was first identified in 1990s, when the artemisinin-heme adducts were identified by mass spectrometry (Meshnick et al., 1991, 1993). Later, artemisinin was verified to alkylate heme *in vitro* via dimethyl ester formation and dematallation (Robert et al., 2002). The heme proteins were subsequently validated as the cellular targets of artemisinin in mice (Robert et al., 2005) and malarial parasites (Creek et al., 2008). Furthermore, sarco/endoplasmic reticulum Ca^2+^-ATPase (SERCA)/PfATP6 (Eckstein-Ludwig et al., 2003), translational controlled tumor protein (TPCP) (Eichhorn et al., 2013), and glutathione *S* transferase (GST) (Lisewski et al., 2014) were independently classified as artemisinin-interactive non-heme proteins in malarial parasites.

Recently, artemisinin has been discovered to be a potential therapeutic agent for ameliorating type 1 diabetes because of its ability to promote the conversion of pancreatic glucagon-producing α cells to insulin-secreting β cells in rats. Specifically, artemisinin binds to the molybdenum (Mo^2+^)-carrying gephyrin to activate the gamma-aminobutyric acid A receptor (GABA_A_R) and inhibit the aristaless related homeobox (ARX), ultimately leading to augmented β cell proliferation, enhanced insulin secretion, and improved glucose homeostasis (Li et al., 2017). Two years ago, artemisinin was elucidated to bind to the heme-containing cytochrome *c* anchored on the mitochondrial respiratory chain complexes in mice, which increases the levels of adenosine monophosphate (AMP) and the oxidized form of nicotinamide adenine dinucleotide (NAD), but decreases those of adenosine triphosphate (ATP) and the reduced form of NAD (NADH). The ultimate outcome is that AMP-activated protein kinase (AMPK), silent information regulator 2 type 1 (SIRT1), and peroxisome proliferator-activated receptor gamma co-activator 1 alpha (PGC-1α) are activated, thereby promoting mitochondrial biogenesis, maintaining telomere integrity, and extending lifespan in yeast and mice (Wang et al., 2015a,b).

There has evidence to associate the artemisinin-conjugated proteins with weight reduction, in which artemisinin was observed to bind to the mitochondrial heme protein cytochrome *c*1 (CYC1) and non-heme protein NADH dehydrogenase ubiquinone flavoprotein 1 (NDUFV1) (Gao et al., submitted). As authors noticed, artemisinin actually also binds cytosolic proteins such as GST and triacylglyceral lipase (TAGL), suggesting a multi-target interaction of artemisinin with cellular proteins.

## Artemisinin Targets Receptors Impacting on Signaling Cascades

Although artemisinin preferentially alkylates heme proteins, it can also conjugate other proteins in a complex or unknown manner. As described, a peptide fragment consisting of Phe12 to Tyr22 of the N-terminal region of TCTP from *P. falciparum* can be alkylated by an artemisinin-derived probe (Li et al., 2016). Nevertheless, the affected receptor entities by artemisinin have been identified for some non-heme proteins. It was demonstrated that artemisinin attenuates portal hypertension in rodents with hepatic fibrosis by inhibiting the activation and contraction of hepatic stellate cells via farnesoid X receptor (FXR) (Xu W. et al., 2017). Artemisinin was also proven to inhibit nociceptive transmission by downregulating the P2X4 receptors and glial fibrillary acidic proteins in satellite glial cells of the dorsal root ganglia, thus relieving neuropathic pain in the chronic constriction injury rat model (Ying et al., 2017).

The extracellular signal-regulated protein kinase (ERK) pathway has been recently shown to be implicated in the neuroprotective effect exerted by artemisinin. For example, artemisinin was confirmed to protect human retinal pigment epithelial cells from hydrogen peroxide-induced and age-related macular degeneration through the activation of ERK/cyclic-AMP response element-binding protein (CREB) signaling (Chong and Zheng, 2016). Interestingly, activation of the ERK1/2 pathway by artemisinin was shown to have potential in the prevention and treatment of Alzheimer’s disease. In another study, clinically relevant concentrations of artemisinin were demonstrated to protect and rescue neuronal PC12 cells from the cell death induced by amyloid beta-peptide 25–35 (Aβ_25-35_), during which artemisinin was verified to act as a neuronal protectant from Aβ_25-35_ insult via activation of the ERK1/2 pathway (Zeng Z. et al., 2017). Furthermore, the neuroprotective effect of artemisinin was assessed by the exposure of neonatal rats to the neurotoxin isoflurane, and artemisinin was found to exhibit an inhibitory effect on isoflurane-induced neuronal cell death and ameliorate cognitive impairment and memory loss by modulating histone acetylation and signal transduction through the c-Jun N-terminal kinase (JNK) and ERK1/2 pathways (Xu G. et al., 2017).

## Artemisinin Exerts Beneficial Effects Via Anti-Inflammation

The beneficial effects of artemisinin may involve an inflammatory response cascade, although there remains a gap in knowledge between targeted proteins and affected receptors. Indeed, artemisinin was suggested to possess anti-inflammatory and anti-oxidant properties against lipopolysaccharide (LPS)-induced acute lung injury in mice by inhibiting Toll-like receptor 4 (TLR4) signaling, leading to a synchronous decline of tumor necrosis factor alpha (TNF-α), interleukin 1 beta (IL-1β), and interleukin 6 (IL-6) levels (Zhao D. et al., 2017). A protective role of artemisinin on chronic alcohol-induced liver damage was also observed, in which the activation of nuclear factor kappa B (NF-κB) was inhibited and the expression of inflammatory cytokine-inducible nitric oxide synthase (iNOS) was downregulated (Zhao X. et al., 2017).

Furthermore, artemisinin was observed to suppress the receptor activator of nuclear factor kappa-B ligand (RANKL)-induced osteoclastogenesis through inhibition of phospholipase C gamma 1 (PLCγ1)-Ca^2+^-nuclear factor of activated T-cells, cytoplasmic 1 (NFATc1) signaling, thereby preventing ovariectomy-induced bone loss in mice with collagen-induced arthritis (CIA) (Zeng X. et al., 2017). Accordingly, SM934, an analog of artemisinin, was shown to attenuate CIA in mice by suppressing T follicular helper cells and T helper 17 cells (Lin et al., 2017).

## Challenges for Excessive Artemisinin Use Other than Anti-Malaria

**FIGURE 1:**
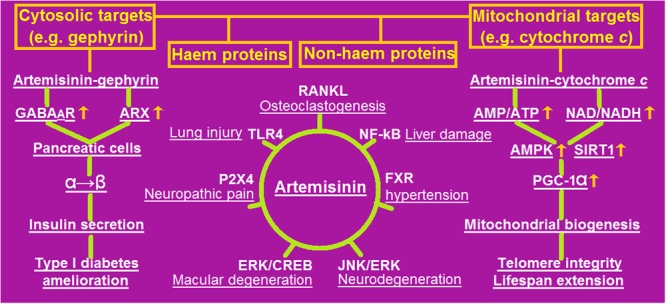
An outline of the most recently revealed pharmaceutical potential with selective signaling mechanisms of artemisinin. Artemisinin can target cytosolic and/or mitochondrial heme and/or non-heme proteins (illustrated in the upper panel), by which many pathogenic processes (underlined) are blocked through specific receptors (depicted in the central panel), including amelioration of type 1 diabetes (in the left panel) and extension of lifespan (in the right panel). AMP, adenosine 5′-monophosphate; AMPK, AMP-activated protein kinase; ARX, aristaless related homeobox; CREB, cyclic-AMP response element-binding protein; ERK, extracellular signal-regulated protein kinase; FXR, farnesoid X receptor; GABA_A_R, gamma-aminobutyric acid A receptor; JNK, c-Jun N-terminal kinase; NAD, oxidized nicotinamide adenine dinucleotide; NADH, reduced NAD; NF-κB, nuclear factor kappa B; PGC-1α, peroxisome proliferator-activated receptor gamma co-activator 1 alpha; RANKL, receptor activator of nuclear factor kappa-B ligand; SIRT1, silent information regulator 2 type 1; TLR4, Toll-like receptor 4.

As a pluripotent drug with increasing clinical value in anti-malarial, anti-tumor, and anti-inflammatory roles, artemisinin seems to be an elixir with metformin- and resveratrol-like effects on human health because they behave as the activators of AMPK and/or SIRT1 in yeast and mice (Wang et al., 2015a,b). The most recently revealed pharmaceutical roles and selective signaling mechanisms of artemisinin were outlined in **Figure 1**.

In contrast to applying a high dose of artemisinin to antimalaria, a low dose of artemisinin is usually chosen for an alternative application. For example, a clinical dose of artemisinin applied for antimalaria is at least 6.7 mg/kg in patients (Zhao et al., 1989), but only 0.25 mg/kg or 260 μM artesunate was used for anti-aging in mice (Wang et al., 2015b), and only 5 μM artemether in zebrafish or 10 μM artemether in mice was used for anti-diabetes (Li et al., 2017). However, long-term and low-dose exposure to artemisinin might induce free-radical scavengers such as the antioxidant enzyme superoxide dismutase (SOD) (Sun and Zhou, 2017), which can destroy the vulnerable endoperoxide bridge structure within artemisinin, thereby leading to the lowered therapeutic inefficiency of artemisinin, a dilemma resembling that of unrestricted antibiotic use.

Importantly, because of the wide-spectrum and non-specific features of artemisinin, some unexpected metabolic dysfunctions or abnormalities, including genotoxicity due to sperm DNA damage (Singh et al., 2015), might emerge upon excessive artemisinin use.

## Artemisinin-Triggered Antioxidative Responses Confer Artemisinin Resistance to Malarial Parasites

Several Southeast Asian countries have currently reported the emergence of malarial parasites that have decreased susceptibility to artemisinin derivatives including artesunate, dihydroxyartemisinin and partner drugs, resulting in the increasing rates of treatment failures (Blasco et al., 2017). Although the resistance mechanisms have not been fully understood, artemisinin-induced enzymatic and non-enzymatic antioxidants that scavenge reactive oxygen species (ROS) might be actively engaged.

Clinically, three homozygous individuals with the inherited deficiency of the antioxidant enzyme glutathione reductase (GR) were shown to provide protection of red blood cells against severe malarial infection, implying a beneficial effect of GR to malarial parasites escaping artemisinin attack (Gallo et al., 2009). A significant increase (2.9-fold) in the level of reduced glutathione (GSH) was determined in the arteether-resistant *Plasmodium vinckei* as compared to arteether-sensitive parasites. Simultaneously, significantly increased activities of GR, GST, and glucose-6-phosphate dehydrogenase (G6PDH) were recorded in resistant parasites. These results indicated that GSH and other antioxidant enzymes may counteract arteether’s antiparasite effects, thereby contributing to the parasites’ resistance to arteether and other artemisinin-based antimalarials (Chandra et al., 2011).

Therefore, we suggest globally assessing the effects of artemisinin on human health, either positive or negative, and also urge avoiding the widespread application of artemisinin to combating the versatile types of human diseases other than malaria. Unless absolutely necessary, artemisinin should be replaced by other therapeutic agents with similar pharmaceutical roles for the treatment of these diseases.

## Author Contributions

Q-PZ and QW wrote the manuscript. D-SY, S-QH, and C-QL critically reviewed the manuscript. All authors have read and approved the final version of the manuscript.

## Conflict of Interest Statement

The authors declare that the research was conducted in the absence of any commercial or financial relationships that could be construed as a potential conflict of interest.
